# Ketone Bodies in the Brain Beyond Fuel Metabolism: From Excitability to Gene Expression and Cell Signaling

**DOI:** 10.3389/fnmol.2021.732120

**Published:** 2021-08-27

**Authors:** Darío García-Rodríguez, Alfredo Giménez-Cassina

**Affiliations:** Department of Molecular Biology, Centro de Biología Molecular “Severo Ochoa” (CBMSO UAM-CSIC), Universidad Autónoma de Madrid, Madrid, Spain

**Keywords:** ketone bodies, brain metabolism, metabolic signaling, neuronal excitability, epilepsy, ketogenic diet, β-hydroxybutyrate, acetoacetate

## Abstract

Ketone bodies are metabolites that replace glucose as the main fuel of the brain in situations of glucose scarcity, including prolonged fasting, extenuating exercise, or pathological conditions such as diabetes. Beyond their role as an alternative fuel for the brain, the impact of ketone bodies on neuronal physiology has been highlighted by the use of the so-called “ketogenic diets,” which were proposed about a century ago to treat infantile seizures. These diets mimic fasting by reducing drastically the intake of carbohydrates and proteins and replacing them with fat, thus promoting ketogenesis. The fact that ketogenic diets have such a profound effect on epileptic seizures points to complex biological effects of ketone bodies in addition to their role as a source of ATP. In this review, we specifically focus on the ability of ketone bodies to regulate neuronal excitability and their effects on gene expression to respond to oxidative stress. Finally, we also discuss their capacity as signaling molecules in brain cells.

## Introduction

Ketone bodies (KBs) were long considered mere by-products of fatty acid breakdown that increase during fasting and other situations of glucose shortage, which can be physiological, including extenuating exercise, or pathological, such as diabetes. In fact, their relationship with starvation and fat mobilization has been studied for several decades (Edson and Leloir, 1936), but their physiological function was not fully understood at the time. KBs were found to be actively oxidized by rat mitochondria isolated from different organs in the 1950s (McCann, 1957). In 1967 KBs were discovered to replace glucose as the major fuel of the brain in situations of prolonged fasting or glucose shortage, being able to supply up to 60% of the energy needs of the brain (Owen et al., 1967). However, the impact of KBs on brain function had been anticipated much earlier, as in the 1920s, it was observed that a low-carb and high-fat diet was successful in treating pediatric epilepsy (McQuarrie and Keith, 1927; Helmholz and Keith, 1933; Keith, 1937).

This diet, which mimicked fasting by forcing fatty acid breakdown by reducing the intake of carbohydrates, was termed the “ketogenic diet,” as it correlated with increased KB [β-hydroxybutyrate (BHB) and acetoacetate (AcAc)] in blood and urine (for a rigorous historical perspective, see Thiele, 2003). With the advent of anticonvulsant drugs, the ketogenic diet was used less and less frequently, although it has regained interest in the last 2–3 decades due to its capacity to treat seizures in pharmacoresistant patients (Thiele, 2003). In this regard, several ketogenic diet formulations (Kossoff et al., 2003; Pfeifer and Thiele, 2005; Neal et al., 2009; Roehl et al., 2019; McDonald and Cervenka, 2020), as well as their underlying protective mechanisms (Lutas and Yellen, 2013; Puchalska and Crawford, 2017; Jensen et al., 2020), are being proposed and evaluated, and it has become an active field of investigation. Since ketogenic diets usually entail a myriad of systemic metabolic changes, their biological effects cannot be exclusively attributed to KBs. However, the significant rise in ketone bodies elicited by ketogenic diets prompted to hypothesize that KBs could have biological roles beyond metabolic fuel.

## Ketone Bodies During Fasting

Before dissecting the biological effects of KBs, it is important to understand the basic biological and physiological context of KBs. For a comprehensive review on ketone body synthesis, release, uptake, and utilization, the readers are referred to recent review articles that have covered this topic extensively (Puchalska and Crawford, 2017; Jensen et al., 2020). Ketone body synthesis, or ketogenesis, takes place mostly in hepatocytes, although other cell types, such as astrocytes in the brain or kidney cells, have also been proposed to synthesize ketone bodies to a lesser extent (Puchalska and Crawford, 2017). On the other hand, ketone body utilization as metabolic substrates, or ketolysis, occurs mainly in the brain, heart, and skeletal muscle (Puchalska and Crawford, 2017). Both ketogenesis and ketolysis are regulated at the whole-body level by the endocrine system, with insulin and glucagon playing a central role in preventing and facilitating ketogenesis and ketolysis, respectively (McGarry and Foster, 1977). Fibroblast growth factor-21 (FGF21), has also emerged as an important hormonal regulator of adaptation to ketotic states during fasting, promoting both ketogenesis in the liver as well as ketone body utilization in peripheral tissues, including the brain (Badman et al., 2007, 2009; Inagaki et al., 2007; Fisher and Maratos-Flier, 2016; Kharitonenkov and DiMarchi, 2017; Katsu-Jiménez and Giménez-Cassina, 2019).

Strikingly, little is still known about the precise underpinnings of regulation of ketone body synthesis and utilization at the biochemical level. The role of the mechanistic target of rapamycin (mTOR) in liver ketogenesis has been put forward as an important regulator of gene expression of the ketogenic and ketolytic machinery through the peroxisome proliferator activated receptor α (PPARα) transcription factor (Sengupta et al., 2010). Sirtuin 3 (SIR3) also contributes to regulating the expression of ketone body-related enzymes (Dittenhafer-Reed et al., 2015). Focusing more specifically on ketogenic and ketolytic enzymes, one of the key aspects regarding ketogenesis is the regulation of its rate-limiting mitochondrial enzyme 3-hydroxy-3-methylglutaryl-CoA synthase-2 (HMGCS2), whose expression is restricted to ketogenic tissues and is tightly regulated by the forkhead transcriptional factor FOXA2 in different physiological contexts (Wolfrum et al., 2004; Von Meyenn et al., 2013). The activity of HMGCS2 is also regulated by post-translational modifications, namely by a phosphoregulatory switch (Grimsrud et al., 2012). Another important enzyme in ketogenesis and ketolysis is acetyl-CoA acetyl transferase-1 (ACAT1), which catalyzes the reversible reaction that breaks acetoacetyl-CoA into two molecules of acetyl-CoA. ACAT1 activity can also be regulated by phosphorylation and subsequent tetramerization (Fan et al., 2016). Of note, the regulation of ACAT1 by phosphorylation in an EGF-dependent manner has been described in the context of tumor growth and its concomitant metabolic rewiring to supply more acetyl-CoA and support cancer cell proliferation (Fan et al., 2016). Therefore, further studies are needed to determine its physiological relevance in the regulation of ketone body metabolism in non-pathological states.

More specifically in the brain, the AMP-dependent kinase (AMPK) pathway has been shown to upregulate ketogenesis in glial cells, by promoting fatty acid breakdown and favoring astrocytic ketone body synthesis (Blázquez et al., 1999). This study suggested that AMPK would drive ketogenesis by promoting fatty acid import into mitochondria. This process would be mediated by phosphorylation and subsequent inhibition of acetyl-CoA carboxylase (ACC), involved in the fatty acid synthesis. AMPK-mediated phosphorylation and inhibition of ACC would prevent the synthesis of malonyl-CoA, which is a known inhibitor of the carnitine-palmitoyl transferase-I (CPT-I), an essential molecule to import fatty acids into mitochondria for further processing. As expected, CPT-I is a key regulator of ketogenesis in astrocytes (Blázquez et al., 1998), and its indirect modulation by the AMPK/ACC axis contributes to fine-tune ketogenesis (Blázquez et al., 1999). Consistently, AMPK can also favor ketone body utilization in cortical neurons in response to FGF21, although the exact mechanisms underlying this effect have not been fully characterized yet, and it is more likely to include additional players (Katsu-Jiménez and Giménez-Cassina, 2019).

Other signaling mechanisms are known to enhance ketone body utilization. For example, the BCL-2 family member BAD, initially described as a pro-apoptotic protein, was later found to modulate metabolic flux in endocrine tissues (Danial et al., 2003, 2008; Giménez-Cassina et al., 2014; Giménez-Cassina and Danial, 2015). Interestingly, BAD phosphorylation acts as a metabolic switch between glucose and ketone body utilization in the brain. Thus, genetic interference with BAD phosphorylation impairs glucose utilization and is linked to increased ketone body-induced mitochondrial respiration both in neurons and astrocytes (Giménez-Cassina et al., 2012b). This phosphoregulatory switch clearly points to a signaling mechanism that modulates the metabolic preference in neural cells, albeit the signals that regulate BAD phosphorylation in the brain to regulate metabolic flux, as well as the exact mechanisms that link BAD phosphorylation with ketone body utilization remain to be fully addressed.

Interestingly, KBs and glucose metabolism are highly intertwined, and they present a reciprocal inhibition. In rat hippocampal brain slices, when both BHB and glucose are present, the former is preferred in glutamatergic neurons for the generation of acetyl-CoA (Valente-Silva et al., 2015). This effect seems to be replicated in humans, as a study in athletes undergoing nutritional ketosis shows a decrease in glycolysis and lactate production while increasing triacylglycerol oxidation in skeletal muscle (Cox et al., 2016). The switch of metabolic substrate preference occurred even with co-ingestion of carbohydrates and normal muscle glycogen, and despite physical workloads that would normally be highly glycolytic. Although still unclear, several mechanisms have been put forward to explain the reduction of glycolytic flow by KBs. Some of the mechanisms proposed include an inhibitory feedback of glycolysis by a high acetyl-CoA/CoA ratio or NADH/NAD+ and the participation of uncoupling proteins (UCP2, UCP3, UCP4; Valente-Silva et al., 2015; Cox et al., 2016; Vallejo et al., 2020). This metabolic shift from carbohydrates to fat metabolism and ketogenesis may even have an effect in increasing longevity and healthspan (Roberts et al., 2017).

In summary, there are still many aspects of the basic regulation of ketogenesis and ketolysis in the brain that need to be studied in depth. This includes the metabolic crosstalk between neurons and glial cells (Fernandez-Fernandez et al., 2012; Vicente-Gutierrez et al., 2019; Jimenez-Blasco et al., 2020), as communication between neurons and glial cells has important physiological repercussions (Liddelow and Barres, 2017; Liddelow et al., 2017). Also, understanding the functional consequences of rewiring the metabolic program in the brain is of high importance. Precisely, the main scope of this review is to cover the molecular underpinnings that link KBs with their biological impact in the brain beyond their role as a fuel, including fine-tuning neuronal excitability, regulation of gene expression, and novel findings that suggest their potential contribution to modulation of signaling networks.

## Ketone Bodies Regulating Neuronal Firing Rates

**Figure 1:**
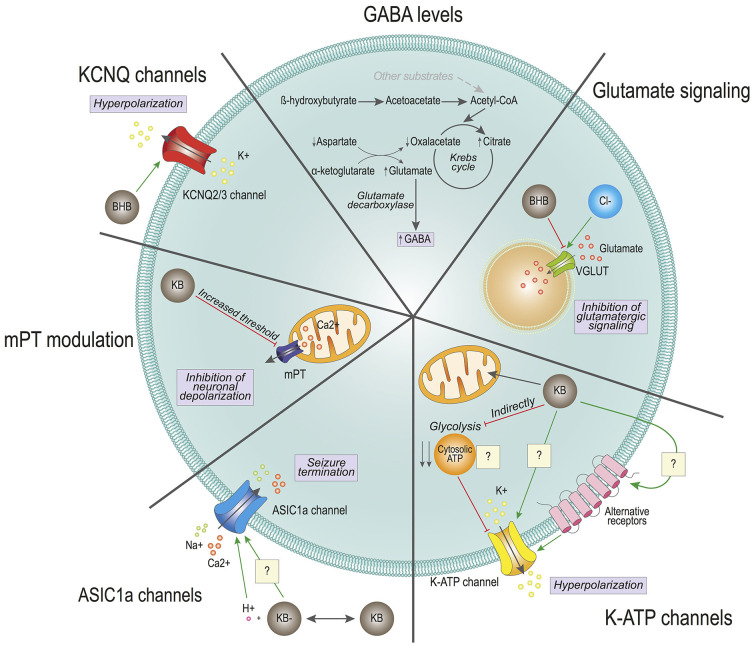
Effects of ketone bodies on cell excitability. The proposed mechanisms for ketone bodies’ (KBs) action on neuronal excitability are depicted. GABA levels: KB β-hydroxybutyrate (BHB) and acetoacetate are converted into Acetyl-CoA at a faster rate than with other substrates, which enters the Krebs cycle reducing the levels of oxaloacetate. To replenish the Krebs cycle, aspartate is converted to oxaloacetate, generating high levels of glutamate. Through the glutamate decarboxylase of GABAergic neurons, glutamate is converted into GABA, increasing the intracellular GABA pool. Glutamate signaling: BHB competes with chloride (Cl-) for the allosteric binding site of the vesicular glutamate transporter (VGLUT). The competition reduces the levels of glutamate inside the vesicles and reduces glutamatergic signaling. K-ATP channels: Ketone bodies (KBs) enter directly into the mitochondria, without generating cytosolic ATP. The lack of cytosolic ATP could provoke the activation of potassium ATP-sensitive (K-ATP) channels, causing the hyperpolarization of the cell. K-ATP channels may also be modulated directly by KBs or indirectly through the activation of alternative receptors. ASIC1a channels: KBs generate a local decrease in pH, which activates the acid sensing ion channel (ASIC1a). These channels participate in seizure termination. KBs may also directly modulate the ASIC1a. KCNQ2/3 channels: BHB directly activates KCNQ channels, which generate a potassium current. This potassium current causes the hyperpolarization of the cell. KBs may also regulate neuronal excitability by participating in mitochondrial permeability transition (mPT) and subsequent oscillations in cytosolic calcium levels.

Given the clinical application of ketogenic diets to treat refractory epilepsy, it would be logical to expect that KBs could exert their therapeutic effect by directly reducing neuronal firing rates (Figure 1). In this regard, one of the very first studies on brain metabolism and the effect of ketogenic diets showed that shifting the source of ATP from glucose to KBs could result in increased ATP:ADP ratios. The study suggested that this raise in the available energy levels could help maintain neuronal “stability” (i.e., resting state), thus reducing the frequency, duration, and/or intensity of depolarization events (DeVivo et al., 1978).

Also, other studies have focused on changes in the levels of neurotransmitters that could explain the reduction in excessive electric activity observed in seizures. One study showed that there was an accumulation of gamma-amino butyric acid (GABA) in synaptic terminals of rats fed a ketogenic diet (Erecińska et al., 1996). Both glucose and ketone body metabolism lead to acetyl-CoA synthesis that feeds the TCA cycle for NADH synthesis and subsequent oxidation in the electron transport chain and ATP synthesis. However, the chain of biochemical reactions that converge in acetyl-CoA synthesis is different in both cases. While glucose undergoes glycolysis in the cytosol to provide pyruvate and subsequently acetyl-CoA, which is oxidized in the mitochondria, KBs provide acetyl-CoA through direct oxidation inside this organelle. Both glucose and KBs provide two molecules of acetyl-CoA per molecule. However, ketone body metabolism yields a rapid high-abundance of acetyl-CoA in comparison to glucose. Overfeeding of the Krebs cycle by KBs reduces the levels of oxaloacetate, which is replenished with the transamination of aspartate through the aspartate transaminase. This reaction requires α-ketoglutarate that is transformed into glutamate, which is then catalyzed in GABAergic neurons into GABA by the enzyme glutamate decarboxylase. Therefore, it was hypothesized that the balance of intermediate metabolites could be responsible for favoring the biochemical synthesis of GABA. Increased secretion of the major inhibitory neurotransmitter of the nervous system could contribute to modulate excessive neuronal firing. And although it was not clear that the GABA levels were overall increased in the whole brain (Yudkoff et al., 2001), the same group performed flux studies that suggested that there could be an increased rate of glutamine and GABA synthesis (Yudkoff et al., 2005). Noteworthy, an increase in the levels of GABA in cerebrospinal fluid has been detected in children treated with a ketogenic diet, thus further strengthening the relevance of ketogenic diets in neurotransmitter balance, particularly GABA (Dahlin et al., 2005). Interestingly, the modulation of GABAergic signaling by KBs is also supported in other animal experimental models, as pharmacological blocking of GABA_B_ receptor abolishes the protective effect of BHB seizure-like activity in *Drosophila melanogaster* (Li et al., 2017). However, KBs do not seem to interact directly with postsynaptic receptors, at least in the hippocampal and entorhinal cortex regions in rats (Thio et al., 2000). Further research will be needed to determine whether the impact of KBs on GABAergic signaling is due mostly to an increase in the intracellular content of GABA, or if there is additional interaction between KBs and other molecular pieces involved in GABAergic neurotransmission. Furthermore, it will be important to ascertain whether this modulation in GABA levels is due to KBs exclusively, or if it is rather derived from the ketogenic diet-elicited systemic metabolic adaptation.

Another mechanism through which KBs may limit the release of the excitatory neurotransmitter glutamate is based on the vesicular glutamate transporters (VGLUTs). VGLUTs are necessary to uptake glutamate in synaptic vesicles for subsequent release. Interestingly, this process is dependent on a low concentration of chloride (Naito and Ueda, 1985). Further studies revealed that chloride is a direct allosteric activator of VGLUTs, and that the ketone body AcAc directly competes for the allosteric docking site of chloride (Juge et al., 2010). In this study, chloride could confer maximal activation of VGLUT2 at 4 mM, and this effect could be drastically counteracted by AcAc with a half-maximal concentration (IC_50_) of 0.5 mM (Juge et al., 2010), which is compatible with physiological circulating levels of AcAc reached during prolonged fasting in adult individuals, up to 1 mM (Garber et al., 1974). Considering that KB levels in brain tissue are usually slightly lower than in circulation (Owen et al., 1967; Cahill, 2006), these results suggest that by limiting glutamate uptake into synaptic vesicles, KBs may moderately decrease the amount of excitatory neurotransmitter glutamate released into the synaptic cleft, thus preventing excessive neuronal firing responsible for seizures.

In line with modulation of neuronal excitability by KBs, several groups have reported direct or indirect effects of ketogenic diets and/or KBs modulating the activity of different ion channels. These events could account for alterations in neuronal firing frequency and intensity. The group of Gary Yellen elegantly showed that both BHB and AcAc reduce firing rates in neurons of the dentate gyrus by favoring the opening state of the potassium ATP-sensitive (K-ATP) channels, which can be blocked by intracellular ATP (Ma et al., 2007). Importantly, additional work showed that opening of K-ATP channels may contribute to modulate neuronal polarization after firing events, and ketone body-induced K-ATP channel opening could be an important contributor to mediate the protective effect of KBs against epileptic seizures (Tanner et al., 2011). This was further demonstrated in a mouse genetic model that has enhanced ketone body metabolism in the brain and is naturally resistant to chronically and acutely induced seizures (Giménez-Cassina et al., 2012b; Martínez-François et al., 2018). In this mouse model, genetic ablation of the Kir6.2 subunit of the K-ATP channel significantly restored sensitivity to seizures, thus supporting a role for K-ATP channels in mediating the effects of KBs in neuronal excitability (Giménez-Cassina et al., 2012b). The mechanism through which KBs modulate the opening of K-ATP channels is not fully clear yet. Glucose metabolism yields both mitochondrial and cytosolic ATP, while ATP derived from ketolysis is generated exclusively in mitochondria. The working hypothesis that was proposed for ketone body regulation of K-ATP channels is that the cytosolic pool of ATP generated from glycolysis could be reducing their open probability, whereas switching to ketolytic metabolism would lead to reduced cytosolic ATP, in turn releasing the inhibition of the K-ATP channel opening (Ma et al., 2007). Of note, glycolytic rates are highly increased in neurons during neuronal stimulation, which could account for metabolic regulation of the K-ATP channels (Díaz-García et al., 2017). Nonetheless, the development of genetically encoded sensors, with higher accuracy and sensitivity, will hopefully contribute to addressing the contribution of ATP compartmentalization to the activity of the K-ATP channels (Koveal et al., 2020). In addition, K-ATP channels can be regulated by several intracellular signaling pathways (Mironov and Richter, 2000), and KBs may also modulate other signaling pathways (see below). Therefore, KBs may regulate the opening of the K-ATP channels employing multiple and diverse mechanisms.

Acid-sensing ion channels (ASIC) have also been postulated to mediate the effects of KBs on neuronal excitability, although their role is still controversial, and their link with KBs is not fully understood. It has been observed that seizures can reduce brain pH from 7.35 to 6.8 through lactic acid production, CO_2_ accumulation, and other mechanisms, and acidosis is also a known inhibitor of seizures, likely through multiple mechanisms (Somjen, 1984). ASIC1a is a proton-gated Na^+^ and Ca^2+^ ion channel that can be activated in response to acidic pH (Yermolaieva et al., 2004), and it has been shown to participate in seizure termination by activating hippocampal inhibitory interneurons, which exhibited larger H+-gated densities than excitatory pyramidal neurons (Ziemann et al., 2008). This was supported by genetic disruption of the *Asic1a* gene or by pharmacologic inhibition of ASIC1a, both of which increased seizure severity in rodents. Consistently, overexpressing *Asic1a* had the opposite effect, suggesting that ASIC1a forms part of a feedback inhibition circuit that controls seizure severity (Ziemann et al., 2008). Along this line, the duration of seizure activity was shorter in wild-type than *Asic1a*^−/−^ mice, and in wild-type mice seizures were less likely to progress to tonic-clonic seizures and death. However, ASIC1a would not be responsible for ketone body-mediated reduction in the onset of seizures, which is supported by the fact that *Asic1a* disruption did not affect seizure threshold, the latency to seizure onset, or initial seizure severity (Ziemann et al., 2008).

This hypothesis linking KBs and ASIC1a has also been challenged by other studies. First, recent studies have shown that ASIC1a inhibition by the non-specific ASIC antagonist amiloride is protective in a rat model of epileptic seizures (Liang et al., 2015). Second, KBs have been recently shown to inhibit ASIC1a channels, but the precise mechanism and the relevance of these channels in epileptic contexts remains an open question (Zhu et al., 2019).

It could be argued that ASIC1a imparts differential effects when it comes to seizure onset and termination. Another possibility to take into account is that ASIC1a is expressed in several neuronal populations, including excitatory and inhibitory neurons. Of note, the aforementioned studies were performed in different models, including species (mouse vs. rat) and pro-convulsant treatment (kainate/pentylenetetrazole vs. lithium-pilocarpine). Moreover, the studies performed *in vitro* to delineate the impact of KBs on ASIC1a could only be performed at very high concentrations of KBs, within ranges close to dangerous ketoacidosis, and at acidic pH (Zhu et al., 2019). Noteworthy, oscillations in brain pH have a clear impact on brain electrical activity (Yuen et al., 2017). One could speculate that KBs, due to their acidic chemical nature, may lead to local pH lowering in the vicinity of ASIC1a, thus contributing to seizure termination (Yuen et al., 2017). However, studies carried out in mice and rats have not been able to detect a reduction in brain pH, thus questioning this hypothesis (Davidian et al., 1978; DeVivo et al., 1978; Al-Mudallal et al., 1996).

For all these reasons, it is difficult to compare studies and draw conclusions. ASIC1a channels could be proposed as new targets for the development of seizure-controlling drugs. However, if and how KBs impinge on ASIC1a function, their relationship and functional relevance is not clear yet and deserves further investigation.

The work of Rho and collaborators showed an additional mechanism through which KBs may regulate neuronal excitability. They showed that KBs directly regulate mitochondrial permeability transition (mPT), thus affecting the regulation of intracellular calcium levels (Kim et al., 2015). MPT consists of increased permeability of the mitochondrial inner membrane in face of a variety of physiological and pathological stimuli (Bernardi et al., 2015; Giorgio et al., 2018). Short, transient increases in mPT can help regulate the levels of intracellular Ca^2+^, thus participating in neuronal depolarization (Bernardi et al., 2015; Giorgio et al., 2018). Precisely, it was shown that KBs could directly increase the threshold for mPT, which in turn reduced the frequency of seizures in experimental mouse models of epilepsy (Kim et al., 2015). In addition, sustained mPT can lead to neuronal cell death due to high cytosolic Ca^2+^ concentration, as well as the release of other pro-apoptotic factors from mitochondria (Bernardi et al., 2015; Giorgio et al., 2018). Therefore, and in light of these results, KBs could also contribute to preserving neuronal cell viability after an epileptic crisis by preventing sustained mPT and subsequent activation of cell death, ultimately protecting neurons from seizure-induced cell death.

Another mechanism through which KBs directly impacts neuronal excitability also involves K+ channels, namely the KCNQ channels (Manville et al., 2020). KCNQ2-5 channels, and more specifically KCNQ2 and KCNQ3 channels, are responsible for the so-called M-currents, which are hyperpolarizing currents that contribute to reducing neuronal firing frequency (Wang et al., 1998). Interestingly, a recent study expressing human KCNQ2/3 on *Xenopus laevis* oocytes showed that BHB can effectively activate the KCNQ2/3 heterotetrameric channel at physiologically relevant concentrations (~0.1 mM) by directly interacting with a tryptophan residue located on a transmembrane segment of the KCNQ3 subunit, thus eliciting neuronal hyperpolarization (Manville et al., 2020).

Using also *X. laevis* oocytes expressing human neurotransmitter receptors, it was found that the KBs acetone and BHB had differential effects on the regulation of GABA, NMDA and glycine receptors at physiologically relevant concentrations for BHB (up to 5 mM; Pflanz et al., 2019). These results were not in agreement with previous findings in a similar experimental model (Yang et al., 2007), although it is important to note that the concentration range used in both studies were significantly different (in the Yang study the concentrations of BHB were much higher, ranging from 10 to 100 mM, thus being outside of physiological concentrations achieved by prolonged fasting and/or ketogenic diets). In addition, no clear mechanism was demonstrated for these effects.

While future studies will help confirm the relevance of many of these findings in the mammalian brain, these results reinforce the notion that KBs have a broad array of non-metabolic biological effects that directly impinge on neurotransmission and neuronal excitability (Figure 1).

## Ketone Bodies and Gene Expression

**Figure 2:**
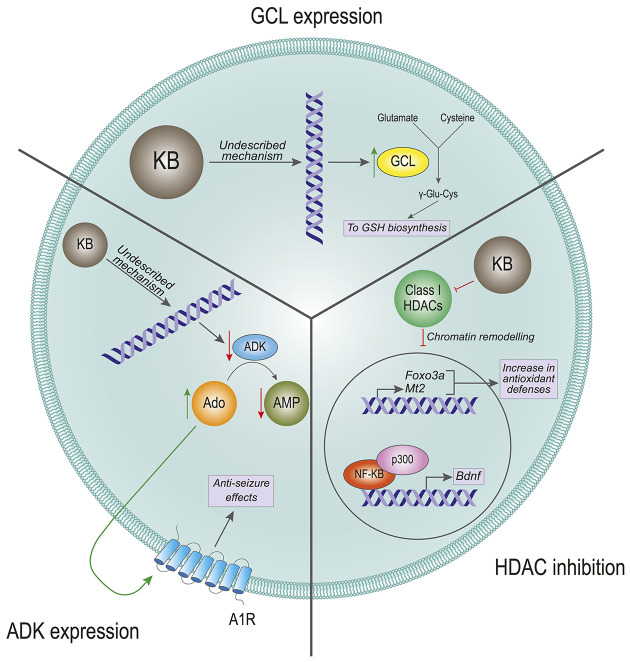
Effects of ketone bodies on gene expression. The proposed mechanisms for the effect of Ketone Bodies (KBs) on gene expression are presented. Glutamate-cysteine ligase (GCL) expression: KBs increase the transcription of the GCL gene, which is the rate-limiting enzyme in the glutathione (GSH) biosynthesis. The incremented expression of GCL increases the levels of GSH, which in turn leads to a rise in antioxidant defenses. HDAC inhibition: KBs are inhibitors of the class I histone deacetylases (HDACs). The inhibition of HDACs provokes a remodeling in the chromatin structure that leads to increased expression of the antioxidant-related genes Foxo3a and Mt2, and to an increased expression of the Bdnf gene mediated by NF-κB and p300. ADK expression: KBs reduce the expression levels of the adenosine kinase (ADK) gene. This transcriptional inhibition favors high levels of adenosine (Ado) that activate the adenosine 1 receptors (A1R). The activation of these receptors have anti-seizure effects on the cell by reducing firing rates.

Among other biological functions, compelling evidence has shown that KBs have the ability to regulate gene expression (Figure 2). One of the first experimental approaches supporting such ability was provided by the group of Manisha Patel, who showed that rats fed a ketogenic diet exhibited increased mitochondrial levels of reduced glutathione (GSH), and increased overall ratio of reduced-to-oxidized glutathione (GSH:GSSG) in the hippocampus (Jarrett et al., 2008). This was achieved through upregulation of protein levels and subsequent increased activity of glutamate-cysteine ligase (GCL), the rate-limiting enzyme in glutathione synthesis (Jarrett et al., 2008). Considering that epileptic seizures cause cellular damage through augmented oxidative stress (Liang et al., 2000; Liang and Patel, 2006), the ability of ketogenic diets to upregulate the glutathione system could confer protection against seizure-induced brain damage. This still did not explain protection against seizures by ketogenic diets *per se*, but at least, by reducing seizure-induced brain damage, it could prevent further neuronal loss and subsequent seizing events by disturbances in neuronal circuitries, as redox control is essential for brain functioning.

An important question arising is: how can ketogenic diets regulate the levels of glutathione synthesis enzymes? Follow-up work showed that ketogenic diets also induce nuclear accumulation of the NF E2-related factor (NRF2) transcription factor (Milder et al., 2010), which is a master regulator of the cellular antioxidant machinery (Yamamoto et al., 2018). At the time, this was attributed to the fact that ketogenic diets induce a mild, transient bout of oxidative stress that could in turn activate the NRF2 pathway as a response (Milder et al., 2010). In addition, another study showed later that BHB can activate the transcription of the antioxidant-related genes *Foxo3* and *Mt2* by directly inhibiting class I histone deacetylases (HDAC) in human embryonic kidney cell lines, as well as in kidney sections from mice treated with BHB (Shimazu et al., 2013). Thus, inhibition of HDACs by BHB would directly contribute to chromatin remodeling and subsequent changes in the gene expression profile. Noteworthy, a study performed in 2006 had already proposed that BHB could alleviate oxidative damage in excitotoxic insults directly applied in the rat striatum (Mejía-Toiber et al., 2006). The mechanism the authors proposed did not include changes in gene expression, but enhanced mitochondrial function that would reduce the production of free radicals.

The investigation on the role of KBs in modulating gene expression was further expanded by other groups. Previous studies had described that physical exercise upregulates the expression of Brain-Derived Neurotrophic Factor (BDNF) in the central nervous system, albeit the exact underlying mechanisms have been widely debated (Sleiman and Chao, 2015). BDNF has important implications in neuronal cell survival and proliferation, which means that modulation of BDNF by cell signaling could have profound implications in neuronal physiology (Mitre et al., 2017). A 2016 study showed that exercise in mice induced an increase in BHB, which in turn would lead to upregulation of *Bdnf* gene expression in the hippocampus (Sleiman et al., 2016). Consistent with previous work (Shimazu et al., 2013), this effect was shown to be mediated by the inhibitory effect of BHB on HDAC, thus leading to activation of the *Bdnf* gene promoter (Sleiman et al., 2016). These findings were further confirmed and complemented by additional work that proposed that BHB could also upregulate the expression of *Bdnf* in rat cortical neurons by modulating the redox balance as well as chromatin remodeling and the activity of additional transcription factors (Marosi et al., 2016).

Ketone body-triggered changes in gene expression intuitively suggest long–term adaptive modifications, as opposed to immediate responses mediated by modulation of neuronal excitability. However, changes in the gene expression profile elicited by KBs may also contribute to influencing fast responses to neurotransmitters. This is the case of ketogenic diet-regulated purinergic signaling. Ketogenic diets in mice were found to downregulate the expression of the enzyme adenosine kinase in the brain (Masino et al., 2011). This enzyme catalyzes the phosphorylation of adenosine to adenosine-monophosphate (AMP), which is an important intracellular signaling molecule and also the precursor of ATP. Reduction in adenosine kinase levels leads to the accumulation of adenosine, which can be transported to the extracellular milieu and act directly on adenosine A_1_ receptors (A_1_R). Interestingly, adenosine modulation of A_1_R has a potent anti-epileptic effect (Dunwiddie and Worth, 1982; Etherington and Frenguelli, 2004). In fact, genetic and pharmacologic modulation of adenosine kinase and A_1_R demonstrated that upregulation of adenosine signaling mediates some of the antiepileptic effects of ketogenic diets (Masino et al., 2011). Importantly, whether the effects of the ketogenic diet on adenosine kinase expression are directly mediated by KBs or by additional ketogenic diet-derived mechanisms remains to be determined.

Altogether, these results reinforce the notion that KBs impact neuronal physiology through a complex interplay of multiple mechanisms. In summary, many of these processes would imply direct and indirect actions of ketone body-mediated changes in gene expression (Figure 2).

## Ketone Bodies: A Role in Cell Signaling?

**Figure 3:**
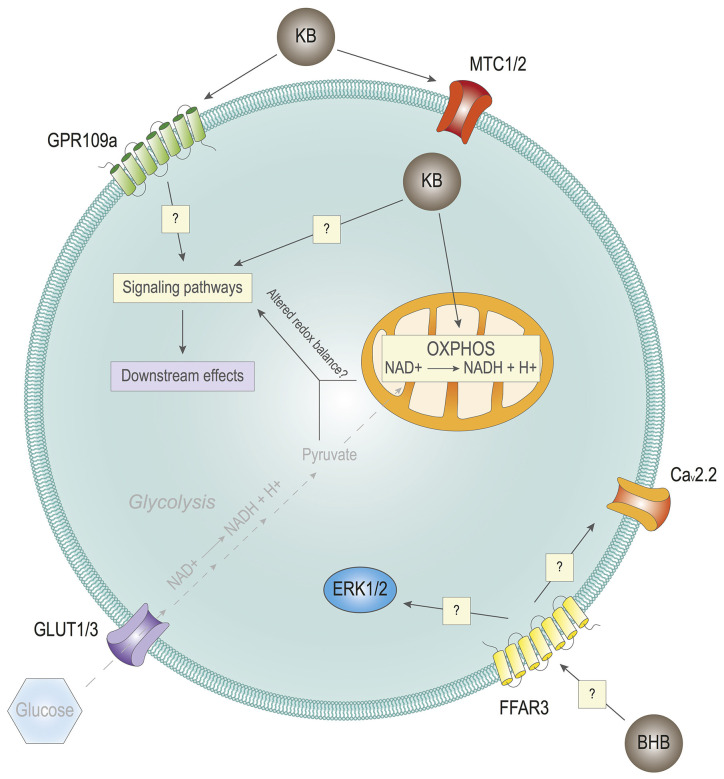
Effects of ketone bodies on cell signaling. Hypothetical impact of Ketone bodies (KB) on cell signaling. KB may impact cell signaling through their extracellular receptors GPR109a and/or FFAR3, having an impact on intracellular cell signaling. KB may also impact cell signaling by entering cells through the monocarboxylate transporters (MTCs) 1/2. Inside the cell, in combination with reduced or absent glycolysis due to very low levels of glucose, KB may alter the redox balance of the cell, also with potential consequences in cell signaling. In turn, the alterations in the signaling pathways of the cell lead to different downstream effects with biological outcomes.

As discussed above, KBs can elicit immediate responses by modulating ion channels, as well as long-term adaptive responses, by conditioning gene expression. Are there additional mechanisms through which cells can sense KBs and elaborate adequate responses to them? Can KBs influence the activity of cell signaling networks? We have previously observed that KBs can actually modulate cell signaling pathways in cultured mouse cortical neurons (Katsu-Jiménez and Giménez-Cassina, 2019). Further pilot experiments combining kinase activity profiling and phosphoproteomics confirm that exposure to BHB affects the activation status of several protein kinase networks in primary cortical neurons (Katsu-Jiménez, García-Rodríguez, and Giménez-Cassina, unpublished data). This is consistent with results that show that exposure to AcAc activates extracellular signal-regulated kinase (ERK) signaling in skeletal muscle cells, albeit the exact mechanism is not completely clear (Zou et al., 2016). Therefore, and in light of these results, it appears that KBs can modulate cell signaling cascades (Figure 3). The question is: how?

Several plasma membrane receptors can respond to select metabolites, such as lactate, succinate, or fatty acids, among others (He et al., 2004; Hirasawa et al., 2005; Bozzo et al., 2013; Lauritzen et al., 2014). Of note, the G protein-coupled receptor (GPCR) for niacin, also known as GPR109a/HCA2/HM74A/PUMA-G, can be activated in response to physiological concentrations of BHB (Taggart et al., 2005). GPR109a is particularly enriched in neutrophils, adipocytes, and macrophages (Maciejewski-Lenoir et al., 2006). Initial studies did not show high expression of GPR109a in the brain (Maciejewski-Lenoir et al., 2006). It was later found that some hypothalamic neurons could express GPR109a, and that its activation may control endocrine regulation by modulating ERK1/2, namely the growth hormone signaling axis (Fu et al., 2015a). Another study found that there is an increased expression in GPR109a levels in the brain of Parkinson’s disease patients, specifically in microglia (Wakade et al., 2014). Interestingly, it was later observed that the GPR109a expression in microglial cells was increased in *in vivo* experimental models of neuroinflammation, which is a common feature of Parkinson’s disease and other neurodegenerative pathologies (Fu et al., 2015b). Furthermore, BHB-induced GPR109a activation significantly contributed to mitigating neuroinflammation and subsequent microglial activation (Fu et al., 2015b). In fact, GPR109a activation has been previously shown to exert anti-inflammatory effects (Digby et al., 2012).

In light of these effects of GPR109a, and considering the biological role of KBs as its endogenous ligand, a question arises as to the physiological meaning that links KBs with inflammation control. Prolonged fasting, which leads to a drastic reduction in glycemia, induces ketone body production. Interestingly, sustained hypoglycemia has been associated with increased inflammatory states (Ratter et al., 2017). Therefore, it could be hypothesized that KBs not only provide an alternative fuel during fasting but also contribute to toning down the concomitant activation of the immune system through GPR109a. This is consistent with a recent study that shows that short–term exposure to a ketogenic diet in mice contributes to lowering systemic inflammation (Goldberg et al., 2020). Importantly, GPR109a expression has been seen to be increased in microglial cells in pro-inflammatory contexts (Wakade et al., 2014). Whether GPR109a can be temporarily expressed in other cell populations of the brain under specific circumstances, the exact signaling mechanisms and its functional significance remain to be elucidated. Of note, GPR109a is not the only mediator of the ability of KBs to modulate the immune system, as BHB can actually attenuate NLRP3-mediated inflammation in immune cells (Youm et al., 2015). Therefore, if and how KBs can modulate neuroinflammation by regulating microglial activity is still an open question.

Another membrane receptor that could participate in KBs-dependent signaling is the free fatty acid receptor 3 (FFAR3/GPR41). At first, this receptor was thought to be activated by short chain fatty acids (SCFA), mainly acetate and propionate (Won et al., 2013). Nonetheless, the concentrations of these metabolites that are needed to activate FFAR3 seem to be too high to be achieved in the plasma of nonruminating mammals. In this context, BHB has proven to interact with FFAR3 in sympathetic neurons, although it remains to be elucidated whether BHB interacts as an agonist or as an antagonist with the receptor. On the one hand, Kimura et al. (2011) found that SCFAs induce the activation of sympathetic neurons in mice mediated by FFAR3. 10 mM BHB causes opposite effects, acting as an FFAR3 antagonist. FFAR3 inhibition downregulates ERK1/2 signaling, which in turn reduces catecholamine release. On the other hand, research carried out by Won et al. (2013) proves that 10 mM BHB activates the FFAR3 receptor in sympathetic neurons of rats. This activation ultimately leads to the inhibition of N-type Ca^2+^ channels (Ca_v_2.2) mediated by Gα_i/o_ proteins. The contradictory findings of these two groups may be explained by differences in the animal models or by the behavior of BHB as a partial agonist. A partial agonist may be confounded as an antagonist in some scenarios. Furthermore, Won et al. (2013) show that the activation of FFAR3 by BHB is less potent than that caused by SCFAs, but reaches the same efficacy. In addition to this debate, there is evidence that butyrate, a metabolite closely related to BHB, activates FFAR3 in the anterior pituitary of rats (Miletta et al., 2014). Nonetheless, the physiological relevance of these results should be interpreted with caution, as 10 mM BHB is a very high concentration that might not reflect what can happen in a healthy subject, even after prolonged fasting.

KB-mediated extracellular signaling is of high relevance in the regulation of food anticipation and time adaptation to feeding. A study found that liver-derived ketone bodies during fasting could promote food anticipation activity in mice (Chavan et al., 2016), which suggests the involvement of liver-brain signaling axis. It was later found that the monocarboxylate transporter-1 (MCT1), the carrier responsible for liver export of ketone bodies to the bloodstream, was required to connect ketogenesis with food anticipation, as genetic ablation of *Mct1* in mouse hepatocytes significantly reduced food anticipation (Martini et al., 2021). However, cell-type-specific genetic deletion of *Mct1* in either neurons or astrocytes did not have any impact on food anticipation (Martini et al., 2021). Considering that neurons predominantly express MCT2 (Pierre and Pellerin, 2005), the effect of KBs on neurons could still be mediated by neuronal uptake. Another possibility is that KBs do not need to be uptaken to regulate food anticipation. In this case, their effect could be mediated extracellularly, through the aforementioned receptors GPR109a or FFAR3. In contrast, another study found that silencing of MCT1 with adenoviral vector-mediated delivery of shRNA in the hypothalamic area of the rat increases food intake (Elizondo-Vega et al., 2016). In this work, the effects of MCT1 knockdown were attributed to lactate transport, and not BHB. However, it is important to consider that the latter study was carried out in rats, not mice, and the experiments were performed with *ad libitum* access to food, as opposed to the former study that included restricted feeding (Martini et al., 2021). In any case, it is clear that the crosstalk between the brain and the periphery is key to ensure proper whole-body metabolic homeostasis. In addition, the expression of MCTs is tightly regulated (Pierre and Pellerin, 2005), also by circadian rhythms (Martini et al., 2021). This highlights the relevance of understanding the coordination between ketone body synthesis, release, and uptake to ensure a timely regulation of KBs function.

Along this line, once KBs enter cells through their corresponding MCT (in the brain, MCT1 predominantly in astrocytes, and MCT2 in neurons; reviewed in Pierre and Pellerin, 2005; Puchalska and Crawford, 2017; Jensen et al., 2020), there are several intracellular mechanisms through which they could hypothetically impact signaling networks. First, several kinases are known to be regulated by select metabolites, such as diacylglycerols or phosphoinositide derivatives (Ubersax and Ferrell, 2007). Therefore, it could be speculated that KBs, or derived metabolites, could directly interact with and modulate the activity of signaling-related proteins. Additionally, KBs may also modulate signaling networks through indirect mechanisms. Changes in nutritional states that prompt a metabolic shift from glucose to ketone body utilization upon prolonged fasting lead to alterations in the redox balance, including variations in cytosolic and mitochondrial NAD(P)/NAD(P)H ratios (Veech et al., 2017; Corkey and Deeney, 2020). This is expected as glucose utilization yields both cytosolic and mitochondrial NADH equivalents, through glycolysis and TCA cycle, respectively; whereas ketone body oxidation takes place exclusively in the mitochondrial compartment.

Precisely, this compartmentalization of redox equivalents could account for changes in cell signaling, partly mediated by thioredoxins and other cellular antioxidant systems (Ren et al., 2017; Miller et al., 2018). To cite some examples, the Akt pathway can respond directly to changes in the redox balance in leukemia and lymphoma cell lines (Pelicano et al., 2006) and AMPK can also be regulated by a thioredoxin-1-mediated redox switch in the mouse heart, through oxidation of two cysteine residues (Shao et al., 2014). Noteworthy, these studies were carried out in non-neuronal cells. AMPK signaling is well known to reprogram metabolic flux to adapt to oscillations in ATP:ADP ratios (González et al., 2020), and it is therefore consistent that it can also respond to metabolic-derived fluctuations in the redox balance as a result of a metabolic shift. As mentioned above, AMPK can drive changes in neuronal metabolism, favoring ketone body utilization (Katsu-Jiménez and Giménez-Cassina, 2019). Altogether, it is suggested that KBs could thus participate in fine-tuning cellular signaling in multiple ways.

Noteworthy, this shift in the redox balance happens in physiological conditions upon the transition between feeding and fasting periods, when hyper- or normo-glycemia are alternated with hypoglycemia and a subsequent rise in ketone body production. It would be interesting to evaluate if KBs, in a normoglycemic milieu, could still induce a shift in the redox balance that could contribute to modulating signaling networks. Importantly, the aforementioned work by Rho and collaborators showed that KBs may still have clear biological effects on mPT even in normoglycemic conditions (Kim et al., 2015), which underlines the relevance of investigating the impact of KBs in relation to glucose concentration.

In any case, the regulation of cell signaling by KBs has not been studied in depth yet. Further studies will be necessary to understand if and how KBs play a role in cell signaling, and to what extent (Figure 3).

## Discussion

KBs display a broad array of biological effects in the brain beyond their role as metabolic fuels. It is important to bear in mind that some of the biological effects described above that might be attributed to KBs have only been observed in ketogenic diet-fed experimental animals, but a direct action imparted by KBs has not been fully confirmed yet. Moreover, the metabolic rates in terms of fatty acid oxidation and ketogenesis in rats and mice are different, which may well also account for the diverse biological effects observed using different experimental models, as ketotic levels might not be comparable (Puchalska and Crawford, 2017). From a basic point of view, understanding their contribution to the modulation of neuronal function can provide new layers of regulation of neuronal excitability, thus adding up to the complexity of networks and circuits in the brain. From a translational point of view, their importance is underscored by the use of ketogenic diets to treat pharmacoresistant epilepsy, as well as potentially other neurological and rare metabolic disorders for which ketogenic diets are currently being evaluated (Paoli et al., 2014; Dahlin et al., 2015; Heussinger et al., 2018; Oonthonpan et al., 2019; Wang et al., 2020). Unfortunately, the extreme composition of ketogenic diets makes it non-palatable and difficult to tolerate by many patients, who develop gastrointestinal complications (Cai et al., 2017), ultimately compromising compliance. In addition, recent studies have alerted of potential side effects (Cai et al., 2017), including an increase in serum lipidemia with concomitant increased risk of cardiovascular alterations (Zamani et al., 2016), higher risk of bone mass reduction (Simm et al., 2017), long-term alterations in the immune system (Goldberg et al., 2020), or increased probabilities for cardiac fibrosis (Xu et al., 2021). Because of these potential adverse effects, and the difficulty to adhere to the diet, different dietary formulations are being proposed to achieve a therapeutic effect with a more tolerable nutritional intervention (Dallérac et al., 2017; Kossoff and Cervenka, 2020). Furthermore, the anti-seizure efficacy in patients treated with ketogenic diets is not always replicated in experimental rodent models, which hampers research on the underlying molecular mechanisms (Holmes, 2008), This is probably due to biological differences in metabolic rates and in cellular responses to KBs between humans and rodents, as well as to the fact that ketosis is tightly controlled in humans to avoid dangerous situations, whereas mice and rats fed a ketogenic diet are allowed to eat *ad libitum*. This becomes even more difficult considering the aforementioned differences in metabolic rates between rats and mice (Puchalska and Crawford, 2017). Nonetheless, understanding how KBs work, as well as other ketogenic diet-derived metabolites, and gaining insight into the molecular impact of KBs in neuronal physiology, could significantly contribute to identifying novel therapeutic targets in which we can benefit from the diet, but without all the accompanying adverse effects.

One of the main questions is whether the therapeutic effects of ketogenic diets can be purely attributed to KBs or to other factors. KBs have direct biological effects, as discussed above. There is a long-standing debate on whether KBs are directly responsible for the protective effects of the ketogenic diet to prevent epileptic seizures. There are several studies addressing the administration of BHB, AcAc, or acetone in diverse models of epilepsy, either chemically or genetically induced. In fact, in some cases, direct administration of KBs may have potent biological outcomes (Likhodii et al., 2003; Nielsen et al., 2019; Pérez-Liébana et al., 2020; Si et al., 2020), although their anti-seizure effect has been variable depending on the experimental model, which probably accounts for the heterogeneous etiology of epileptic seizures, and also the KBs may only act on some determined types of seizures (Simeone et al., 2018; Si et al., 2020). But in addition to the clear biological effects of KBs *per se*, it must be considered that the increase of KBs in circulation, either *via* fasting or through ketogenic diets, entails profound systemic metabolic changes (Kennedy et al., 2007; Longo and Mattson, 2014; De Cabo and Mattson, 2019). Some of these changes include lower glycemia, changes in the endocrine profile, and alterations in the circulating levels of other metabolites, such as amino acids and/or long-, medium- and short-chain fatty acids, with potential additional biological effects through diverse mechanisms (reviewed in Katsu-Jiménez et al., 2017). As an example, and as discussed above, BHB directly upregulates BDNF expression by remodeling chromatin accessibility (Marosi et al., 2016; Sleiman et al., 2016). However, another work had previously shown that lowering glycolytic flux, which physiologically happens in parallel to ketone body utilization, negatively regulates BDNF expression (Garriga-Canut et al., 2006). This was attributed to subcellular compartmentalization of NAD+ reduction to NADH, which can modulate the assembly of transcriptional complexes (Zhang et al., 2002; Garriga-Canut et al., 2006). The fact that blocking glycolytic flux represses BDNF while increasing ketone body levels upregulates its expression seems contradictory, as ketogenesis is activated as a result of glucose scarcity. Previous reports had already linked BDNF signaling with metabolic rewiring (Burkhalter et al., 2003; Giménez-Cassina et al., 2012a). In light of these findings, and given the importance of BDNF in neuronal development and survival (Mitre et al., 2017), it appears that metabolic oscillations have a profound impact on cellular physiology, and it highlights the complexity of how metabolic networks are tightly intertwined with physiopathological processes. Hence the need to dissect carefully the molecular impact of metabolic shifts and the crosstalk between different metabolic pathways.

## Concluding Remarks

In summary, KBs are fascinating metabolites that exhibit a myriad of biological functions beyond their role as energy fuels, and they constitute an active field of research. There are still many lingering questions as to how they exert their biological effects, and whether they can exert such effects alone or in combination with the concomitant metabolic changes linked to ketone body increase. Understanding in depth their biology will not only provide new layers of regulation of neurophysiological processes highly intertwined with ketone body metabolism but may also contribute to opening up new avenues of research to identify and characterize novel therapeutic targets for neurological disorders.

## Author Contributions

DG-R and AG-C conceived and wrote the manuscript. DG-R prepared all the figures. All authors contributed to the article and approved the submitted version.

## Conflict of Interest

The authors declare that the research was conducted in the absence of any commercial or financial relationships that could be construed as a potential conflict of interest.

## Publisher’s Note

All claims expressed in this article are solely those of the authors and do not necessarily represent those of their affiliated organizations, or those of the publisher, the editors and the reviewers. Any product that may be evaluated in this article, or claim that may be made by its manufacturer, is not guaranteed or endorsed by the publisher.

## Abbreviations

KB: ketone bodies
BHB: β-hydroxybutyrate
AcAc: acetoacetate
BDNF: brain-derived neurotrophic factor
HDAC: histone deacetylase
AMPK: AMP-dependent kinase
mPT: mitochondrial permeability transition
MCT: monocarboxylate transporter.
